# Vitamin D status and type 2 diabetes incidence in Finnish adults—a longitudinal survey and register-based study using standardized serum 25-hydroxyvitamin D data

**DOI:** 10.1007/s00394-025-03889-2

**Published:** 2026-01-24

**Authors:** Folasade A. Adebayo, Suvi T. Itkonen, Tuija Jääskeläinen, Tommi Härkänen, Kevin D. Cashman, Maijaliisa Erkkola, Christel Lamberg-Allardt

**Affiliations:** 1 Department of Food and Nutrition, University of Helsinki, P.O. Box 66, FI-00014, Helsinki, Finland; 2 Institute for Health and Welfare, P.O. Box 30, FI-00271, Helsinki, Finland; 3 Cork Centre for Vitamin D and Nutrition Research, School of Food and Nutritional Sciences, University College Cork, T12 E31 Cork, Republic of Ireland

**Keywords:** Vitamin D, Type 2 diabetes, Fortification, General population, Cohort, Follow-up, Risk factor

## Abstract

**Purpose:**

Improved vitamin D status (serum 25-hydroxyvitamin D; S-25(OH)D) has been recorded among Finnish adults. Whether this change lowers the risk of diabetes is unknown. Our study investigated the associations between improved vitamin D status and long-term type 2 diabetes (T2D) incidence in the Finnish adult population.

**Methods:**

Finnish adults aged ≥ 30 years (n = 3014) in a longitudinal setting (Health 2000/2011 surveys, H2000/H2011) were followed for 8.2 years (2011–2019), and T2D cases were derived from national registers. Multivariable-adjusted Cox regression models were used to assess the associations between S-25(OH)D and incident T2D. S-25(OH)D concentration measurements were standardized according to the Vitamin D Standardization Program protocol.

**Results:**

During the follow-up, 214 T2D incident cases (7%) were recorded. Median (IQR) S-25(OH)D concentration increased from 47.6 (37.1; 57.4) nmol/L in H2000 to 67.2 (60.0; 74.8) nmol/L in H2011. Participants with new incident T2D had lower median (IQR) S-25(OH)D concentrations in H2011 than non-cases [63.97 (57.3; 73.8) vs. 67.2 (60.0; 74.8) nmol/L]. No association between S-25(OH)D concentrations in H2011 and T2D incidence in the whole study sample. Subjects with insufficient vitamin D status (< 50 nmol/L) in H2000 who were in the lowest tertile of S-25(OH)D in H2011 (< 62.8 nmol/L) had a higher T2D risk (adjusted HR 1.61 [95% CI 1.03–2.51]) than those in the highest tertile (> 71.6 nmol/L). Longitudinal changes in S-25(OH)D did not associate with T2D incidence in the whole study sample.

**Conclusion:**

The improved vitamin D status was not associated with T2D incidence in the general follow-up sample. However, vitamin D insufficiency in 2000 (< 50 nmol/L) combined with having S-25(OH)D in the lowest tertile in 2011 (< 63 nmol/L) increased T2D risk. Maintaining an optimal vitamin D status may be protective against T2D.

**Supplementary Information:**

The online version contains supplementary material available at 10.1007/s00394-025-03889-2.

## Introduction

Vitamin D plays multiple roles in the body, including calcium regulation and bone metabolism, modulation of the immune system, and potential protection against autoimmune disorders [1, 2]. These occur through its biologically active metabolite, 1,25-dihydroxyvitamin D (1,25(OH)_2_D), which is needed for the expression of hundreds of genes in a large number of cells of different organs [2, 3]. Hence, vitamin D plays an important role as a mediator of immune-related disorders by e.g. regulating cell proliferation and stimulating insulin secretion in the pancreas [2, 3].

Type 2 diabetes (T2D) is a chronic metabolic disease among adults manifesting as high blood glucose concentrations because the body either becomes insulin-resistant or the pancreas does not produce insulin in sufficient quantities [4]. In Finland, with a population of 5.5 million inhabitants, approximately 400 000 persons are affected by T2D [5]. T2D has been associated with insufficient vitamin D status (serum 25-hydroxyvitamin D [S-25(OH)D] < 50 nmol/L) in many observational studies [1].

Like other countries, especially those at northern latitudes, insufficient vitamin D status has been acknowledged as a problem in Finland (60-70^o^N) since the twentieth century [6]. This is largely due to insufficient sun-induced vitamin D_3_ synthesis in the skin, arising from limited ultraviolet B irradiation, particularly during the long winter season [6]. However, the situation has changed in Finland, with increases in vitamin D intakes since the implementation of the vitamin D food fortification policy in 2003. The recommendation was to add 0.5 µg/100 g vitamin D to all fluid milk products and respective lactose-free milk-, soy-, and cereal-based drinks and 10 µg/100 g to fat spreads [7], and the fortification levels were doubled in 2010, i.e., 10 µg and 20 µg, respectively [8]. Also, with increases in vitamin D supplement use over the years, vitamin D status in the general Finnish population has improved greatly [9–12]. This has been illustrated by the observed increases in S-25(OH)D based on two national surveys of adults in Finland—Health 2000 (H2000) and Health 2011 (H2011)—which straddle the period prior to and after the implementation of food fortification policies [9].

However, it has remained unclear whether the improved vitamin D status (observed in H2011), arising from the increased vitamin D intake from the nationwide vitamin D fortification policy and supplementation use over the years, has had any implications on health outcomes such as decreasing disease incidences in the population. Following the improvement in vitamin D status between 2000 and 2011, when the national surveys were respectively conducted, a follow-up period from 2011 onwards is expected to provide valuable insights into the impact of improved vitamin D status on the risk of T2D. Hence, the aim of this study was to investigate associations between the improved vitamin D status [S-25(OH)D in H2011] and long-term T2D incidence in the Finnish adult population.

## Methods

### Study design and population

**Fig. 1:**
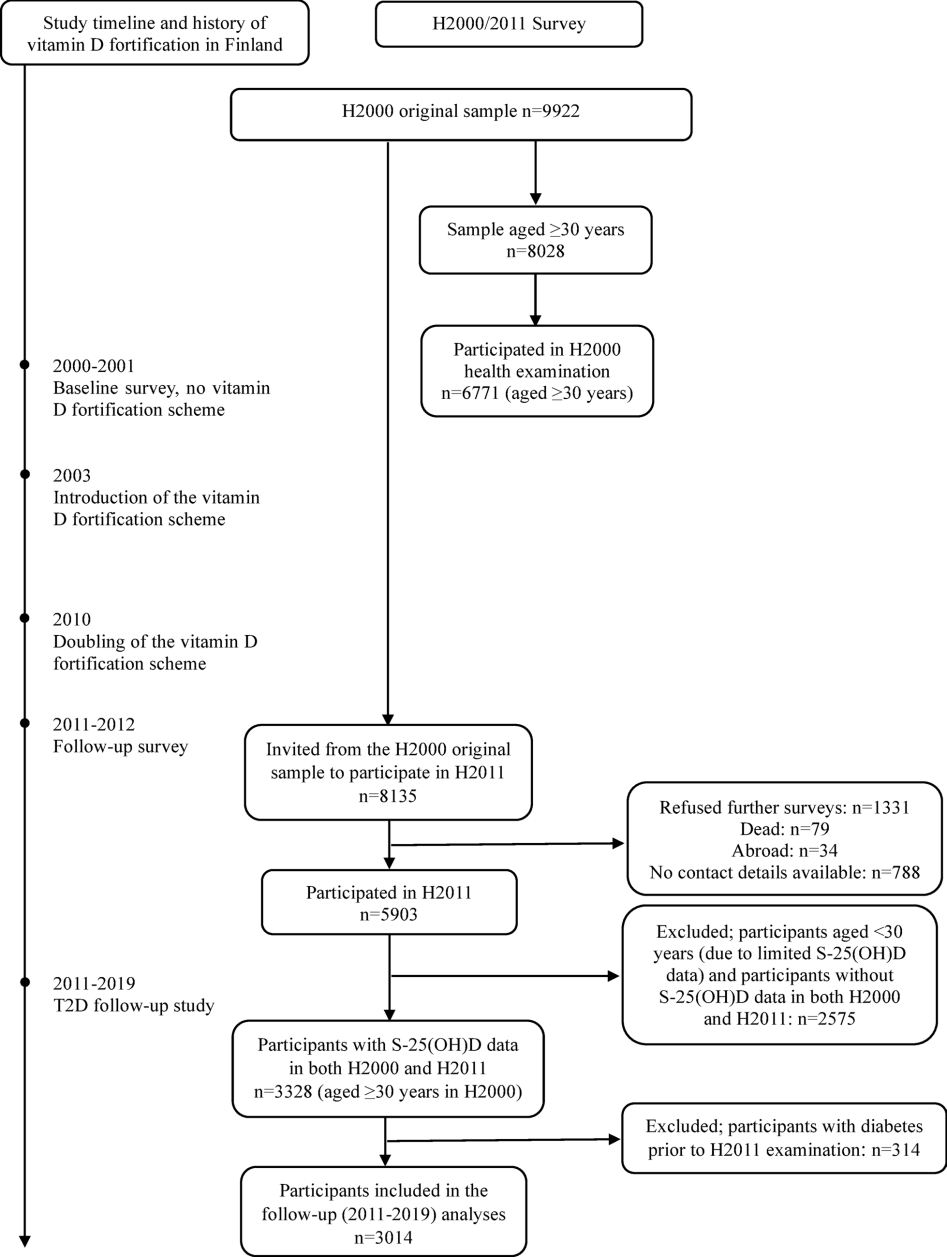
Flow chart of participants in this follow-up study showing timelines and history of vitamin D fortification in Finland. Abbreviations: *H2000* (Health 2000), *H2011* (Health 2011), *S-25(OH)D* (serum 25-hydroxyvitamin D), *T2D* (Type 2 Diabetes)

The H2000/H2011 Survey is a longitudinal cohort survey representing Finnish adults, living in mainland Finland [13, 14]. The H2000/H2011 Survey was conducted by the Finnish Institute for Health and Welfare (THL) through questionnaires, interviews, and a comprehensive health examination. The baseline survey, H2000, was carried out between 2000 and 2001 in a two-stage stratified cluster sample drawn from the national population register in Finland [13]. Altogether, the invited original sample in 2000 consisted of 9922 subjects. Out of the 8028 subjects aged ≥ 30 years, 6771 participated in the health examination. The follow-up survey, H2011, was conducted between 2011 and 2012 [14]. All subjects of the original H2000 sample who were alive, were living in Finland in 2011, and did not decline further contacts were invited to participate in 2011 (n = 8135); 5903 participated in H2011. In this study, analyses included participants aged ≥ 30 years in H2000 with available data on S-25(OH)D concentration in both H2000 and H2011 and who did not have T2D prior to the H2011 examination (n = 3014) (Fig. 1). Subjects aged < 30 years were not included because of limited available data on S-25(OH)D concentration.

### Participants’ characteristics

Information on age and sex was obtained from the sampling frame, while sociodemographic (education and marital status) and lifestyle (smoking status, physical activity, and alcohol consumption) data were obtained through interviews and self-administered health questionnaires [13, 14]. During comprehensive health examinations, weight, height, and waist circumference were measured, and body mass index (BMI) (i.e., weight (kg)/height (m^2^) in kg/m^2^) was calculated. BMI was thereafter defined into four categories: underweight (< 18.5 kg/m^2^), normal weight (18.5–24.9 kg/m^2^), overweight (25.0–29.9 kg/m^2^), and obesity (≥ 30.0 kg/m^2^) according to WHO [15]. Educational status was categorized into three groups (low [basic education i.e., primary and lower secondary education]; middle [upper secondary and vocational education]; high [bachelor’s degree or higher]) and marital status into two groups (married or cohabitation; others, i.e., divorced/separated, widow, and single). Leisure-time physical activity was classified into three categories (inactive [reading, watching television, and other inactive activities]; moderate physical activity [walking, cycling, and moving in other ways at least 4 h per week]; active/vigorous physical activity [active/exercise at least 3 h per week or vigorously active several times per week]). Current daily smoking status included two groups (no [not at all] and yes [daily and occasional smoking]), and alcohol consumption was also categorized into two groups (no [non-drinker and stopped drinking] and yes [current alcohol consumption]). Blood pressure was measured two times with a 2-min interval based on auscultatory method, and the mean value was used. Participants’ characteristics were based on information and measurements in H2000.

### Serum 25(OH)D and other laboratory measurements

In both H2000 and H2011, participants were asked to fast at least 4 h prior to blood sampling during health examination, and serum was separated and stored at −70 °C until analysis [9, 14]. In H2000, the blood samples were drawn between September and March, whereas they were drawn between August and December in H2011 [9]. The season of blood sampling was then classified as winter (November-March) and other season (April–October). The total S-25(OH)D concentrations from both surveys were standardized according to the Vitamin D Standardization Program (VDSP) by means of a certified liquid chromatography-tandem mass spectrometry method (LC–MS/MS) at the University College Cork, Ireland [9, 16]. The LC–MS/MS method used has been certified under the Centers for Disease Control and Prevention Vitamin D Standardization Certification Program [17]. The VDSP protocol is described in detail elsewhere [16, 18].

In this study, vitamin D status was defined according to the Institute of Medicine (IOM) S-25(OH)D thresholds [19], which are employed by both the Nordic and Finnish Nutrition Recommendations [20, 21]. S-25(OH)D < 30 nmol/L was defined as deficient; S-25(OH)D 30 to < 50 nmol/L as insufficient; S-25(OH)D ≥ 50 nmol/L as sufficient [19]. Serum fasting glucose, triglyceride, total cholesterol, high-density lipoprotein (HDL) and low-density lipoprotein (LDL) cholesterol concentrations were analysed within 6 months of blood sampling using standard methods [13].

### T2D incidence and follow-up time

Incident cases of diagnosed diabetes, based on the International Classification of Diseases, 10th revision codes E10-E14, were identified through linkages to the Care Registers for Social Welfare and Health Care and the Registers of the Social Insurance Institution (Kela) for (1) entitlements to specially reimbursed medications due to specific chronic conditions and (2) purchases of prescribed medicines, using the nationwide individual unique identification number as the identity link, governed by THL. Thereafter, we identified incident T2D cases based on age at onset of diabetes, i.e., ≥ 40 years in 2011 [22], which was applicable to all subjects in this study (age at onset of diabetes ≥ 43 years). The T2D incidences were identified up to the year 2019 from the register data. In this study, the follow-up period was limited to the years 2011 to 2019 to explore the impact of the increased vitamin D fortification scheme in 2010. Follow-up time was defined as the number of days from the date of the H2011 examination to the dates of T2D incidence, death, or end of follow-up (i.e., 31 December 2019), whichever occurred first. The median follow-up time from H2011 until the year 2019 was 8.2 years (range 0.01–8.40).

### Statistical methods

All analyses were performed using the Statistical Analysis System for Windows, Enterprise Guide 8.3 (SAS Institute Inc., Cary, NC, USA), accounting for the sampling design by using the Survey procedures. The inverse probability weights were used in all analyses to correct for the effects of non-response [23, 24]. Participants’ characteristics were presented as weighted medians with interquartile range (IQR) for continuous variables and weighted percentages for categorical variables. We analysed S-25(OH)D as categorical variables in terms of status (i.e., insufficient [< 50 nmol/L] and sufficient [≥ 50 nmol/L]), and tertiles. For S-25(OH)D in H2011, the tertiles were as follows: lowest [< 62.8], middle [62.8–71.6], and highest [> 71.6]. Similarly, the tertiles for S-25(OH)D in H2000 were lowest [< 40.3], middle [40.3–54.4] and highest [> 54.4]. Longitudinal changes in S-25(OH)D concentration were calculated by subtracting the VDSP standardized S-25(OH)D concentration in H2000 from the VDSP standardized S-25(OH)D concentration in H2011 i.e., Changes in S-25(OH)D = S-25(OH)D in 2011 − S-25(OH)D in 2000, in nmol/L. The longitudinal changes in S-25(OH)D were also presented as tertiles (i.e., lowest [< 13.2], middle [13.2–26.0], and highest [> 26.0]). Additionally, we assessed the distribution of higher S-25(OH)D cut-offs in H2011 and H2000 among participants i.e., S-25(OH)D < 75 vs. ≥ 75 nmol/L. The potential confounding covariates (age, sex, baseline S-25(OH)D, season of blood sampling, BMI, educational status, physical activity, and smoking status) were selected based on evidence from the literature and preliminary analyses performed in this study. In addition, a DAGitty directed acyclic graph (DAG) was constructed to guide the selection of the appropriate confounders for adjustment in analysis models (Fig. S1) [25]. Participants with prevalant diabetes before or at H2011 examination were excluded from all analyses. To detect possible selection bias, we assessed the S-25(OH)D concentrations of all H2011 participants according to prevalent diabetes status (i.e., S-25(OH)D available at one time point [in H2000 or H2011] and at two time points [in both H2000 and H2011]. We observed that the median S-25(OH)D concentrations did not differ among samples regardless of number of S-25(OH)D measurement and prevalent diabetes status (Table S1).

Univariable and multivariable cox proportional hazards models were used to calculate hazard ratios (HRs) with 95% confidence intervals (CIs) for the associations between participants’ S-25(OH)D concentration (tertiles), sociodemographic characteristics, and lifestyle characteristics and T2D incidence. The univariable model included T2D incidence as a dependent variable and each independent variable (S-25(OH)D in H2011, age in H2000 [as a continuous variable], sex, educational status, BMI [as a continuous variable], physical activity, and smoking status), one at a time. In the multivariable model, estimating HRs for the association between participants’ S-25(OH)D concentration and T2D incidence, all variables plus baseline S-25(OH)D and season of blood sampling were included simultaneously with the independent variable of interest. We performed tests for effect modification by BMI and the other participants’ characteristics in univariate models by including first-degree interaction terms with these variables and S-25(OH)D.

In assessing the association between S-25(OH)D concentration in H2011 (tertiles) and T2D incidence (new-onset cases vs. non-cases) further, three Cox proportional hazards models were employed to estimate hazard ratios (HRs) with 95% CIs. Model 1 was adjusted for age in H2011 (as a continuous variable), sex, baseline S-25(OH)D (as a continuous variable), and season of blood sampling. Model 2 additionally was adjusted for BMI (as a continuous variable), and Model 3 included the variables in Model 2 plus educational status, physical activity, and smoking status. To examine the impact of baseline vitamin D status (i.e., in 2000) on the association, subgroup analysis was also performed according to S-25(OH)D status in H2000, using cut-offs of < 50 nmol/L (insufficient) and ≥ 50 nmol/L (sufficient). Furthermore, the association between longitudinal changes in S-25(OH)D (tertiles) over 11 years and T2D incidence during the follow-up period was investigated using three similar Cox proportional hazards models as described above. Subgroup analysis according to S-25(OH)D status in H2000 was also conducted. Potential interactions were tested by including interaction terms between S-25(OH)D and each of the covariates in the models. In sensitivity analyses, we repeated the analyses by stratifying the data according to BMI categories. The results of all analyses were considered statistically significant at *p* < 0.05.

## Results

### S-25(OH)D concentration, T2D incidence, and sociodemographic, lifestyle, and metabolic characteristics

This study included 3014 participants aged 30–86 years in H2000, who did not have T2D prior to H2011, and 1689 (55%) of whom were women. A significant increase in median (IQR) S-25(OH)D concentrations from 47.6 (37.1; 57.4) nmol/L in H2000 to 67.2 (60.0; 74.8) nmol/L in H2011 was observed, with 94% and 24% of the participants having S-25(OH)D ≥ 50 nmol/L and ≥ 75 nmol/L, respectively, in H2011 (Table 1, Table S2). In H2011, lower S-25(OH)D concentrations were observed among subjects who had blood sampling in winter 2000, had higher BMI, were less physically active, were smokers, or did not consume alcohol (Table S2). In H2011, the differences by age, educational status, and marital status had disappeared (Table S2).

**Table 1:** S-25(OH)D concentrations and sociodemographic, lifestyle, and metabolic characteristics of participants (in H2000) by incident type 2 diabetes status in 2019, i.e., non-cases vs. cases, n = 3014—non-diabetic participants in both H2000/H2011^1^

	Non-cases (n = 2800)	Cases (n = 214)
**S-25(OH)D concentration in H2011**		
S-25(OH)D) in 2011, Median (IQR)	67.2 (60.0; 74.8)	63.9 (57.3; 73.8)
Vitamin D status, % (n)		
Insufficient (< 50 nmol/L) Sufficient (≥ 50 nmol/L)	6 (177) 94 (2623)	7 (14) 93 (200)
S-25(OH)D ≥ 75 nmol/L: yes/no, % (n)		
S-25(OH)D < 75 nmol/L S-25(OH)D ≥ 75 nmol/L	76 (2126) 24 (674)	79 (167) 21 (47)
S-25(OH)D as tertiles, % (n)		
1st (< 62.8 nmol/L) 2nd (62.8–71.6 nmol/L) 3rd (> 71.6 nmol/L)	32 (892) 33 (942) 35 (966)	43 (90) 28 (60) 29 (64)
S-25(OH)D) in 2000, Median (IQR)	48.1 (37.1; 57.4)	44.5 (35.0; 53.7)
Vitamin D status in 2000, % (n)		
Insufficient (< 50 nmol/L) Sufficient (≥ 50 nmol/L)	56 (1571) 44 (1229)	65 (137) 35 (77)
S-25(OH)D in 2000 ≥ 75 nmol/L: yes/no, % (n)		
S-25(OH)D in 2000 < 75 nmol/L S-25(OH)D in 2000 ≥ 75 nmol/L	96.1 (2690) 3.9 (110)	98.2 (210) 1.8 (4)
S-25(OH)D in 2000 as tertiles, % (n)		
1st (< 40.3 nmol/L) 2nd (40.3–54.4 nmol/L) 3rd (> 54.4 nmol/L)	31.4 (867) 33.7 (949) 34.9 (984)	35.5 (75) 40.2 (87) 24.3 (52)
**Sociodemographic variables**		
Age (years), Median (IQR)	47.0 (38.0; 56.0)	50.0 (42.0; 57.0)
Age (years), % (n)		
30–39 40–49 50–59 60–69 > 70	30 (823) 28 (791) 24 (703) 14 (376) 4 (107)	16 (32) 33 (70) 31 (69) 14 (30) 7 (13)
Sex, % (n)		
Men Women	44 (1199) 56 (1601)	60 (126) 40 (88)
Season of blood sampling, % (n)^2^		
Winter 2000 and 2011 Other season 2000 and 2011 Winter 2000 and other season 2011 Other season 2000 and winter 2011	29 (803) 27 (748) 37 (1020) 8 (229)	28 (59) 25 (53) 38 (84) 9 (18)
Educational status, % (n)		
Low Middle High	29 (753) 35 (971) 36 (1067)	38 (74) 38 (84) 24 (55)
Marital status, % (n)		
Married or cohabitating Others	77 (2170) 23 (621)	74 (160) 26 (53)
**Lifestyle variables**		
BMI (kg/m^2^), Median (IQR)	25.4 (23.1; 28.4)	28.5 (26.4; 31.4)
BMI (kg/m^2^), % (n)		
< 18.5 (underweight) 18.5–24.9 (normal weight) 25.0–29.9 (overweight) ≥ 30.0 (obese)	1 (15) 44 (1246) 40 (1108) 15 (430)	0 (1) 18 (37) 45 (96) 37 (80)
Physical activity, % (n)		
Physical inactivity Moderate physical activity ≥ 4 h/week Active/vigorous physical activity ≥ 3 h/week	21 (573) 57 (1580) 22 (617)	28 (58) 52 (114) 20 (41)
Current smoking, % (n)		
No Yes	75 (2108) 25 (682)	69 (146) 31 (67)
Alcohol consumption, % (n)		
No Yes	14 (363) 86 (2373)	18 (36) 82 (173)
**Metabolic variables**		
Waist circumference (cm), Median (IQR)	89.0 (80.5; 97.5)	98.5 (91.0; 107.0)
Systolic blood pressure (mmHg), Median (IQR)	126.0 (116.0; 139.0)	135.0 (125.0; 146.0)
Diastolic blood pressure (mmHg), Median (IQR)	80.0 (73.0; 88.0)	85.0 (79.0; 92.0)
Total cholesterol (mmol/L), Median (IQR)	5.8 (5.1; 6.5)	6.1 (5.3; 6.9)
Serum LDL cholesterol (mmol/L), Median (IQR)	3.6 (2.9; 4.3)	3.8 (3.2; 4.7)
Serum HDL cholesterol (mmol/L), Median (IQR)	1.3 (1.1; 1.6)	1.2 (1.0; 1.4)
Serum triglyceride (mmol/L), Median (IQR)	1.2 (0.9; 1.6)	1.4 (1.1; 2.2)
Serum fasting glucose (mmol/L), Median (IQR)	5.2 (4.9; 5.5)	5.6 (5.2; 5.9)

^1^Crude n, weighted prevalence, and median. ^2^Winter: November-March; Other season: April–October. Abbreviations: *S-25(OH)D* (serum 25-hydroxyvitamin D), *H2000* (Health 2000), *H2011* (Health 2011), *BMI* (body mass index), *HDL* (high-density lipoprotein), *LDL* (low-density lipoprotein). Missing information: educational status (n = 10), marital status (n = 10), BMI (n = 1), physical activity (n = 31), smoking status (n = 11), alcohol consumption (n = 69), systolic blood pressure (n = 5), diastolic blood pressure (n = 6), total cholesterol (n = 2), serum LDL cholesterol (n = 2), serum HDL cholesterol (n = 2), serum triglyceride (n = 2), serum fasting glucose (n = 2)

S-25(OH)D distribution in H2011 and sociodemographic, lifestyle, and metabolic characteristics of participants in Health Surveys, categorized by T2D incidence during the follow-up period (2011–2019), are detailed in Table 1. At the end of the median follow-up period of 8.2 years (range 0.01–8.40), 214 incident T2D cases were observed. Compared with non-T2D cases, we noted that participants with new incident T2D over the follow-up had modestly, lower median S-25(OH)D in H2011 [67.2 (60.0; 74.8) vs. 63.9 (57.3; 73.8) nmol/L] (Table 1). When the participants were categorized by S-25(OH)D status in H2011, there was no observed difference in T2D incidence between those who were vitamin D insufficient and sufficient (> 50 nmol/L). However, compared with non-cases, T2D incidences were more likely to occur among subjects with S-25(OH)D concentration < 62.8 nmol/L, based on tertiles categories. Moreover, the T2D incidence was higher in participants who were older, men (60%), or less educated, who had higher BMI (82%), or who were less physically active. For all metabolic variables, those who did not have T2D had more optimal median values (i.e., had lower median waist circumference, systolic blood pressure, diastolic blood pressure, total cholesterol, serum LDL cholesterol, serum triglyceride, serum fasting glucose, and higher serum HDL cholesterol concentrations) than T2D cases.

### Association between improved vitamin D status and T2D incidence

**Fig. 2:**
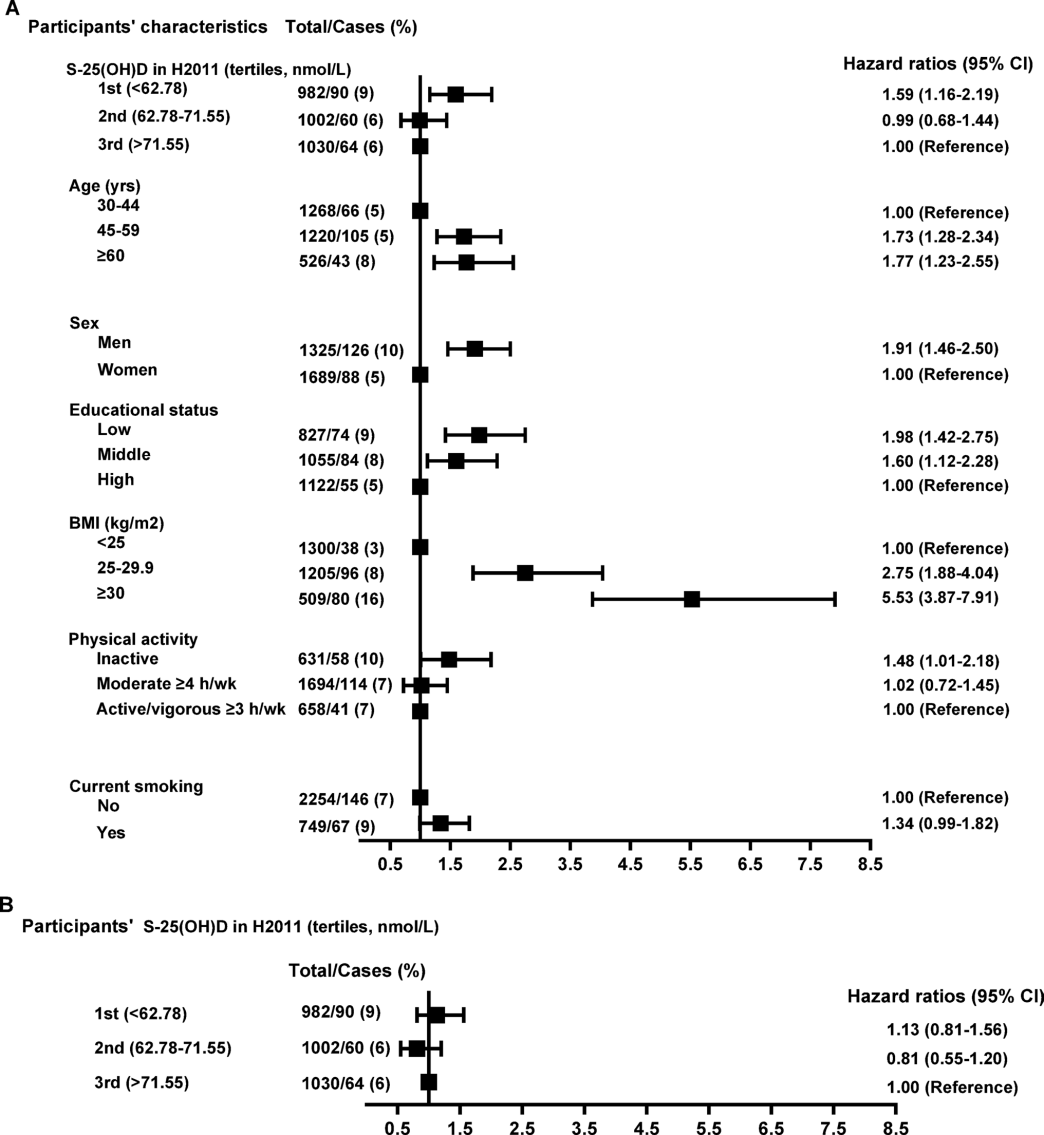
Univariable and multivariable-adjusted hazard ratios (HRs) for associations between participants’ S-25(OH)D in H2011 and relevant covariates (sociodemographic, lifestyle, and vitamin D-related characteristics in H2000) and type 2 diabetes incidence (n = 3014; event = 214) – non-diabetic participants in both H2000/H2011. **A** univariable and **B** multivariable (including all variables shown in Figure A plus baseline S-25(OH)D and season of blood sampling) Cox regression. Crude n and HRs with 95% confidence intervals (CIs). Abbreviations: *S-25(OH)D* (serum 25-hydroxyvitamin D), *H2000* (Health 2000), *H2011* (Health 2011), *BMI* (body mass index)

Table 2 and Fig. 2B present the HRs with 95% CIs for the association between S-25(OH)D in H2011 (as tertiles) and T2D incidence. The highest S-25(OH)D tertile (> 71.6 nmol/L) was used as a reference. In analysis among all participants, no association was observed between S-25(OH)D in H2011 and T2D incidence in all models. In subgroup analysis among subjects who had insufficient vitamin D status [S-25(OH)D < 50 nmol/L] in H2000 (n = 1708, i.e., 57%), compared with participants in the highest tertile, the lowest tertile (S-25(OH)D < 62.8 nmol/L) was associated with 82% increased risk of T2D incidence after adjustment for age (in H2011), sex, baseline S-25(OH)D, and season of blood sampling in model 1 (Table 2). With further adjustment for BMI (model 2) and in the fully adjusted model with additional adjustment for educational status and lifestyle variables (model 3), the increased risk of T2D incidence with S-25(OH)D < 62.78 nmol/L remained (Table 2). An interaction emerged in model 3 between S-25(OH)D and BMI. However, the HRs from sensitivity analysis show no associations by BMI categories (Table S3). No association was seen between S-25(OH)D in H2011 and T2D incidence among subjects whose vitamin D status was sufficient in H2000 (Table 2). Figure 2A demonstrates the associations of confounding factors with T2D incidence. There was no effect modification by BMI on the association between the lowest tertile of S-25(OH)D and T2D risk i.e., compared with participants having normal BMI, HR is 1.28 (95% CI 0.50–3.26) for participants who were overweight and 0.73 (95% CI 0.28–1.89) for those who were obese. We also did not observe any effect modification by other variables for risk of T2D with the lowest tertile of S-25(OH)D.

**Table 2:** Hazard ratios (HRs) with 95% CIs for type 2 diabetes incidence by S-25(OH)D in H2011 and longitudinal changes in S-25(OH)D (tertiles), n = 3014—non-diabetic participants in both H2000/H2011^1^

	n	Incident cases, % (n)	HR (95% CI)		
			Model 1	Model 2	Model 3
**S-25(OH)D in H2011—all subjects**					
All	3014	7 (214)			
1st (< 62.8) 2nd (62.8–71.6) 3rd (> 71.6)	982 1002 1030	9 (90) 6 (60) 6 (64)	1.37 (1.00–1.88) 0.89 (0.61–1.30) 1.00	1.18 (0.85–1.64) 0.82 (0.56–1.21) 1.00	1.13 (0.81–1.56) 0.81 (0.55–1.20) 1.00
**S-25(OH)D in H2011—subjects with insufficient vitamin D status in H2000**					
All	1708	8 (137)			
1st (< 62.8) 2nd (62.8–71.6) 3rd (> 71.6)	755 551 402	11 (77) 7 (37) 6 (23)	1.82 (1.17–2.81) 1.14 (0.64–2.01) 1.00	1.61 (1.04–2.49) 1.08 (0.61–1.92) 1.00	1.61 (1.03–2.51) 1.10 (0.62–1.95) 1.00
**S-25(OH)D in H2011—subjects with sufficient vitamin D status in H2000**					
All	1306	6 (77)			
1st (< 62.8) 2nd (62.8–71.6) 3rd (> 71.6)	227 451 628	6 (13) 5 (23) 7 (41)	0.84 (0.44–1.60) 0.74 (0.44–1.25) 1.00	0.72 (0.36–1.44) 0.66 (0.39–1.12) 1.00	0.60 (0.30–1.23) 0.63 (0.36–1.11) 1.00
**Changes in S-25(OH)D—all subjects**					
All	3014	7 (214)			
1st (< 13.2) 2nd (13.2–26.0) 3rd (> 26.0)	995 1024 995	6 (60) 9 (88) 7 (66)	1.30 (0.84–2.01) 1.55 (1.10–2.19) 1.00	1.12 (0.72–1.75) 1.42 (1.00**–**2.02) 1.00	1.09 (0.70–1.70) 1.37 (0.96–1.97) 1.00

^1^Crude n, hazard ratios (HRs) with 95% confidence intervals (CIs)

Abbreviations: *S-25(OH)D* (serum 25-hydroxyvitamin D), *H2000* (Health 2000), *H2011* (Health 2011), *BMI* (body mass index)

Model 1: adjusted for age in H2011 (as a continuous variable), sex, baseline S-25(OH)D, and season of blood sampling (winter 2000 and 2011, other season 2000 and 2011, winter 2000 and other season 2011, other season 2000 and winter 2011)

Model 2: adjusted for model 1 + BMI (as a continuous variable)

Model 3: adjusted for model 2 + educational status (low, middle, high), physical activity (physical inactivity, moderate physical activity ≥ 4 h/week, active/vigorous physical activity ≥ 3 h/week), and smoking status (no, yes)

### Longitudinal changes in S-25(OH)D concentration and association with T2D incidence over an 8-year follow-up

Longitudinal changes in S-25(OH)D concentration were seen over the 11 years (from H2000 to H2011). A substantial median increase of 19.2 (9.7; 29.6) nmol/L was observed in S-25(OH)D concentrations between H2000 and H2011 (Table S4). When tertile categories of longitudinal changes in S-25(OH)D concentration (i.e., 1st (lowest); 33% [n = 995], 2nd (middle); 34% [n = 1024], and 3rd (highest); 33% [n = 995]) were assessed in relation to baseline concentrations, the observed changes (∆) were inversely proportional to the median baseline concentrations i.e., the participants with lower baseline (bl) S-25(OH)D concentrations had a higher increase and vice versa (1st [bl = 58.2 (50.8; 65.6); ∆ = 5.2 (-0.3; 9.6)], 2nd [bl = 47.6 (40.3; 55.2); ∆ = 19.1 (16.3; 22.5)], and 3rd [bl = 36.1 (29.7; 43.4); ∆ = 34.3 (29.7; 40.3)] nmol/L) (Table S4). Regarding baseline vitamin D status, similarly, the overall change in S-25(OH)D concentrations was higher among participants with insufficient vitamin D status (26.3 [18.0; 34.8] nmol/L) than among those having S-25(OH)D concentrations ≥ 50 nmol/L (10.8 [2.9; 18.4] nmol/L) (Table S4).

In Table 2, HRs with 95% CI for T2D incidence over an 8-year follow-up (i.e., 2011–2019) were estimated by comparing the tertiles of longitudinal changes in S-25(OH)D, using the highest tertile as a reference. Among all participants, the middle tertile of longitudinal changes in S-25(OH)D was associated with a 55% increased risk of T2D incidence in model 1 (adjusted for age in H2011, sex, baseline S-25(OH)D, and season of blood sampling) but not in model 2, with additional adjustment for BMI. Upon full adjustment for all covariates in model 3, with further adjustment for education and other lifestyle variables (physical activity, and smoking) the observed association was not sustained (Table 2). In subgroup analysis among subjects who had insufficient vitamin D status, compared with participants in the highest tertile, the middle tertile of longitudinal changes in S-25(OH)D (13.2–26.0 nmol/L) was associated with increased risk of T2D incidence in all models (Table S5).

## Discussion

In this longitudinal cohort survey and register-based follow-up study, the first to investigate long-term T2D incidence in relation to vitamin D status in the Finnish adult population, a notable increase in S-25(OH)D concentrations was observed among participants between 2000 and 2011, as reported earlier for the whole H2000/H2011 cohort [9]. The crude results showed that S-25(OH)D in H2011 was modestly lower in T2D cases than in non-cases. T2D incidence was higher in participants in the lowest tertile of S-25(OH)D concentration in H2011 (< 63 nmol/L) than in those with concentrations > 72 nmol/L. While no association between improved vitamin D status and T2D incidence was found in the general follow-up sample, an increased risk of T2D was observed among participants with S-25(OH)D < 63 nmol/L who were vitamin D-insufficient in H2000. The association remained after adjustment for several covariates, including BMI, which is a very strong determinant for both S-25(OH)D concentration and T2D incidence. Longitudinal changes, presented as tertiles, did not associate with T2D incidence in the whole study sample after adjustments.

Similar to our findings, compared with those who did not have T2D, a higher prevalence of T2D incidence has been found in participants who were older, less educated, had higher BMI, were less physically active, and were current smokers in a 31-year follow-up study conducted in Finland [26] and in other shorter term prospective cohort studies in other countries [27–29].

The evidence of no association between improved vitamin D status and T2D incidence in our general follow-up sample may be attributed to the fact that the increasing S-25(OH)D status due to vitamin D fortification from 2003 onwards may have changed the course of T2D risk within the 19-year window (2000 to 2019, i.e., H2000 to the end of follow-up). A recent RCT in Finland failed to find any effect of 40 or 80 µg daily vitamin D supplementation on the incidence of T2D in older healthy subjects during a mean 4.2-year follow-up [30]. This is in line with the previous RCTs where the causal relationship between vitamin D status and T2D could not be established because most of the trials, like in the Finnish study, were performed in vitamin D-replete populations (baseline S-25(OH)D ≥ 50 nmol/L) [1, 31]. Hence, this may similarly justify our finding, given that most of the participants (94%) were not vitamin D-insufficient/deficient in H2011. The association between the lowest tertile of S-25(OH)D concentration in H2011 and increased risk of T2D incidence among subjects with insufficient vitamin D status in H2000 suggests that having a higher concentration (> 72 nmol/L) might reduce the risk of T2D. While there was an indication that the association in our study was modified by BMI, such result could not be confirmed through sensitivity analysis. After adjusting for BMI, a known risk factor for T2D [32], we found a 61% increased risk for incident T2D in subjects having 25(OH)D concentrations < 63 nmol/L, in comparison with concentrations > 72 nmol/L, who were vitamin D-insufficient in H2000. Nevertheless, the relatively wide 95% CI width of our results (fully adjusted HR 1.61 [95% CI 1.03–2.51]) indicated uncertainty about the precise magnitude of the association.

Insulin resistance has been linked to obesity and obesity is often associated with a low S-25(OH)D [33]. In our study, 82% of the T2D incident cases were overweight/obese and we found an association between higher BMI and T2D incidence, Hence, the suggestion that higher S-25(OH)D concentration may improve insulin sensitivity and function [33] is reasonable, thus probably reducing risk of T2D in overweight/obese individuals. An increase in T2D incidence with lower 25(OH)D concentrations than in our study has been observed in earlier longitudinal studies with no history of fortification [32, 34, 35]. Among Norwegian adults in an 11-year follow-up study, a higher risk of incident T2D was associated with having S-25(OH)D concentration < 50 nmol/L [34]. Correspondingly, in the longer follow-up of the Young Finns Study, in which data were collected in a different time frame from ours, higher quartiles of mean S-25(OH)D concentration (≥ 52.5 nmol/L; i.e., mean concentrations from youth in 1980 to adulthood in 2007) were associated with reduced T2D risk in adulthood over a 31-year period compared with subjects in the lowest quartile (15–44 nmol/L), even after adjustments [26]. Mechanisms explaining these associations may be linked to the regulatory role of 1,25(OH)_2_D in insulin secretion due to the presence of vitamin D receptors in pancreatic beta cells [33]. As the substrate for production of 1,25(OH)_2_D, sufficient 25(OH)D concentrations thus possibly support maintaining beta cell function, reducing the risk of T2D. Nevertheless, inconsistent and non-causal associations of S-25(OH)D with T2D and prediabetes have also been reported [29, 36]. Moreover, some new findings revealed inverse relationships between 25(OH)D and risk of T2D [37, 38]. A recent systematic review of RCTs regarding clinical practice guidelines showed evidence of an association between vitamin D supplementation and reduced risk of incident diabetes (10 studies: follow-up time = 6 months–5 years) and decreased fasting blood glucose (12 studies: follow-up time = 3 months–5 years) in adults with prediabetes [38].

Regarding longitudinal changes in S-25(OH)D, when compared with the highest tertile, we found an association between the middle tertile (change of 13–26 nmol/L) and T2D incidence, after adjustment for age, sex, baseline S-25(OH)D, and season of blood sampling. However, when BMI and other covariates were introduced to the model the association disappeared. Still, bearing in mind that the observed changes were inversely proportional to the median baseline concentrations, i.e., the participants with lower baseline S-25(OH)D concentrations had a larger increase in S-25(OH)D, the associations observed between the longitudinal changes in S-25(OH)D and T2D incidence were not deemed reliable, as the changes are not analogous. Studies examining longitudinal changes in S-25(OH)D and T2D incidence are rare. Recently in the Tromsø case–control study, such associations were explored among 254 participants [39]. Based on logistic regression models, each prediagnostic 5 nmol/L increase in S-25(OH)D concentrations was associated with 21% lower odds of T2D among women over a 15-year period. In addition, sufficient vitamin D status was inversely associated with T2D incidence among women [39]. In line with other studies, regardless of design [40, 41], baseline S-25(OH)D was observed as a predictor of change in S-25(OH)D concentration over time, with a higher increase in subjects with lower baseline concentrations or insufficient vitamin D status and vice versa. Mechanisms explaining this inverse relationship may include the suggested view that hepatic hydroxylation increases with low 25(OH)D concentrations and decreases when 25(OH)D concentrations are higher [42].

While sufficient vitamin D status has been observed in the majority of the Finnish adult population, our study revealed that at least 34% (n = 982) of the participants in 2011 had S-25(OH)D < 63 nmol/L, 9% of whom had T2D. Low intake of vitamin D-fortified foods or very low status in H2000 may be the underpinning reasons for having a vitamin D status in the lowest tertile in H2011. The differences in S-25(OH)D concentrations in terms of BMI, physical activity, smoking status, and alcohol consumption showed that individual lifestyle factors seem to play unique roles in longitudinal changes in S-25(OH)D concentration. Earlier cohort studies have also reported low concentrations of S-25(OH)D in participants who had higher BMI, were less physically active, and were smokers [32, 43]. Regarding BMI, the present study confirms the well-established inverse association between body weight/BMI and S-25(OH)D concentration because of the volume dilution and/or sequestration of vitamin D in the larger body pool of fat in overweight/obese persons relative to their normal-weight peers [44, 45]. In a previous study on H2000 data, higher S-25(OH)D concentration was found to be associated with healthy lifestyle (e.g., BMI < 25.0 kg/m^2^, being physically active, and no current smoking) and good metabolic health factors [46]. Taken together, our findings alongside previous ones show that maintaining a sufficient vitamin D status in connection to healthy nutrition (including adequate vitamin D intake from fatty fish, vitamin D-fortified dairy and plant-based products, and fat spreads), healthy lifestyle, and weight management is important to protect against T2D.

The main strengths of this study are the longitudinal follow-up of a large H2000/H2011 cohort, a nationally representative population sample, and the use of data on T2D from the relevant nationwide register, which is reliable based on use of diabetes medication. The measurements of S-25(OH)D at two time points (years 2000 and 2011) effectively reflected long-term exposure to vitamin D intake. Moreover, the use of VDSP standardized S-25(OH)D data eliminated concerns regarding accuracy and comparison [47] over the two different time points. Study limitations include possible misclassification of T2D, as we were unable to exclude other types, such as gestational diabetes, that may exist. However, we assumed that nearly all incident cases were T2D because age at onset of diabetes was > 40 years, and it has been reported that the national prevalence of diagnosed type 1 diabetes and other diabetes types is less than 1% [5, 48]. Lack of information on the number of persons that moved abroad during our follow-up study is another limitation. Nevertheless, given the number of persons that moved abroad between 2000 and 2011 (n = 34; Fig. 1), we assumed that the number of such individuals is quite low within 2011–2019 and that their data are not included in our study. Missing data were handled in all analyses by using inverse probability weights, which accounted for non-participation [23]. Despite our comprehensive adjustments for potential confounders and tests for interaction, there was possibility that our results on the association between S-25(OH)D < 63 nmol/L and increased risk of T2D incidence among participants with insufficient baseline vitamin D status could be modified (e.g., by BMI). Bias in terms of different months/seasons of blood sampling may exist despite adjustment. In addition, our results may be influenced by selection biases rising from the exclusion of participants without S-25(OH)D measurements at two time points (years 2000 and 2011) and those with prevalent diabetes, because there is likelihood that descriptive characteristics differ between the included sample and those excluded. Moreover, we could not account for the use of relevant medications, such as lipid-lowering or blood pressure medications. Thus, we cannot rule out the possibility of residual confounding, especially regarding risk factors of T2D, which may contribute to the imprecise magnitude of our results. Given the observational nature of this study, causal inference is not possible with our findings. Nevertheless, longitudinal cohort studies offer a higher level of observational evidence [49]. While our results on associations between vitamin D and T2D should be cautiously interpreted, our findings remain reliable, as they are based on well-designed long-term cohort and register datasets with standardized methods. Additional follow-up data in forthcoming years are required for stronger associative evidence. It is also important that future research examines whether higher S-25(OH)D concentrations, i.e., ≥ 75 nmol/L, without exceeding the safe upper value for S-25(OH)D, are beneficial against T2D incidence. Moreover, the association between longitudinal changes in 25(OH)D and T2D incidence needs further investigation.

## Conclusion

In this study, no association was found between improved vitamin D status and T2D incidence in the general follow-up (2011–2019) sample. However, the study showed that status < 63 nmol/L in 2011 (the lowest tertile) contributed to increased risk for T2D in individuals who were vitamin D-insufficient in 2000. In general, maintaining an optimal vitamin D status may be protective against T2D.

## Supplementary Information

Below is the link to the electronic supplementary material.

Supplementary Material 1

## Data Availability

H2000/2011 data are not publicly available since they include confidential information. The data can be used for research and monitoring of the health and well-being of the population at THL and with collaborators based on collaboration agreement. The data available from the THL Biobank cover those who have participated in the health examination (biological sample available) and can be applied via the THL Biobank in accordance with the Biobank Act and THL Biobank research areas (thl.fi/biobank).
