# Correction: Vitamin D status and type 2 diabetes incidence in Finnish adults—a longitudinal survey and register-based study using standardized serum 25-hydroxyvitamin D data

**DOI:** 10.1007/s00394-026-03950-8

**Published:** 2026-07-11

**Authors:** Folasade A. Adebayo, Suvi T. Itkonen, Tuija Jääskeläinen, Tommi Härkänen, Kevin D. Cashman, Maijaliisa Erkkola, Christel Lamberg-Allardt

**Affiliations:** 1 Department of Food and Nutrition, University of Helsinki, P.O. Box 66, 00014 Helsinki, Finland; 2 Institute for Health and Welfare, P.O. Box 30, 00271 Helsinki, Finland; 3 Cork Centre for Vitamin D and Nutrition Research, School of Food and Nutritional Sciences, University College Cork, T12 E31 Cork, Republic of Ireland

**Correction to: European Journal of Nutrition (2026) 65:34** 10.1007/s00394-025-03889-2

In the original version of this article, the values presented in Tables 1 and 2 are not well aligned.

Tables 1 and 2 should have appeared as shown below.

**Table 1:** S-25(OH)D concentrations and sociodemographic, lifestyle, and metabolic characteristics of participants (in H2000) by incident type 2 diabetes status in 2019, i.e., non-cases vs. cases, n = 3014—non-diabetic participants in both H2000/H2011^1^

	Non-cases (n = 2800)	Cases (n = 214)
**S-25(OH)D concentration in H2011**		
S-25(OH)D) in 2011, Median (IQR)	67.2 (60.0; 74.8)	63.9 (57.3; 73.8)
Vitamin D status, % (n)		
Insufficient (< 50 nmol/L)	6 (177)	7 (14)
Sufficient (≥ 50 nmol/L)	94 (2623)	93 (200)
S-25(OH)D ≥ 75 nmol/L: yes/no, % (n)		
S-25(OH)D < 75 nmol/L	76 (2126)	79 (167)
S-25(OH)D ≥ 75 nmol/L	24 (674)	21 (47)
S-25(OH)D as tertiles, % (n)		
1st (< 62.8 nmol/L)	32 (892)	43 (90)
2nd (62.8–71.6 nmol/L)	33 (942)	28 (60)
3rd (> 71.6 nmol/L)	35 (966)	29 (64)
S-25(OH)D) in 2000, Median (IQR)	48.1 (37.1; 57.4)	44.5 (35.0; 53.7)
Vitamin D status in 2000, % (n)		
Insufficient (< 50 nmol/L)	56 (1571)	65 (137)
Sufficient (≥ 50 nmol/L)	44 (1229)	35 (77)
S-25(OH)D in 2000 ≥ 75 nmol/L: yes/no, % (n)		
S-25(OH)D in 2000 < 75 nmol/L	96.1 (2690)	98.2 (210)
S-25(OH)D in 2000 ≥ 75 nmol/L	3.9 (110)	1.8 (4)
S-25(OH)D in 2000 as tertiles, % (n)		
1st (< 40.3 nmol/L)	31.4 (867)	35.5 (75)
2nd (40.3–54.4 nmol/L)	33.7 (949)	40.2 (87)
3rd (> 54.4 nmol/L)	34.9 (984)	24.3 (52)
**Sociodemographic variables**		
Age (years), Median (IQR)	47.0 (38.0; 56.0)	50.0 (42.0; 57.0)
Age (years), % (n)		
30–39	30 (823)	16 (32)
40–49	28 (791)	33 (70)
50–59	24 (703)	31 (69)
60–69	14 (376)	14 (30)
> 70	4 (107)	7 (13)
Sex, % (n)		
Men	44 (1199)	60 (126)
Women	56 (1601)	40 (88)
Season of blood sampling, % (n)^2^		
Winter 2000 and 2011	29 (803)	28 (59)
Other season 2000 and 2011	27 (748)	25 (53)
Winter 2000 and other season 2011	37 (1020)	38 (84)
Other season 2000 and winter 2011	8 (229)	9 (18)
Educational status, % (n)		
Low	29 (753)	38 (74)
Middle	35 (971)	38 (84)
High	36 (1067)	24 (55)
Marital status, % (n)		
Married or cohabitating	77 (2170)	74 (160)
Others	23 (621)	26 (53)
**Lifestyle variables**		
BMI (kg/m^2^), Median (IQR)	25.4 (23.1; 28.4)	28.5 (26.4; 31.4)
BMI (kg/m^2^), % (n)		
< 18.5 (underweight)	1 (15)	0 (1)
18.5–24.9 (normal weight)	44 (1246)	18 (37)
25.0–29.9 (overweight)	40 (1108)	45 (96)
≥ 30.0 (obese)	15 (430)	37 (80)
Physical activity, % (n)		
Physical inactivity	21 (573)	28 (58)
Moderate physical activity ≥ 4 h/week	57 (1580)	52 (114)
Active/vigorous physical activity ≥ 3 h/week	22 (617)	20 (41)
Current smoking, % (n)		
No	75 (2108)	69 (146)
Yes	25 (682)	31 (67)
Alcohol consumption, % (n)		
No	14 (363)	18 (36)
Yes	86 (2373)	82 (173)
**Metabolic variables**		
Waist circumference (cm), Median (IQR)	89.0 (80.5; 97.5)	98.5 (91.0; 107.0)
Systolic blood pressure (mmHg), Median (IQR)	126.0 (116.0; 139.0)	135.0 (125.0; 146.0)
Diastolic blood pressure (mmHg), Median (IQR)	80.0 (73.0; 88.0)	85.0 (79.0; 92.0)
Total cholesterol (mmol/L), Median (IQR)	5.8 (5.1; 6.5)	6.1 (5.3; 6.9)
Serum LDL cholesterol (mmol/L), Median (IQR)	3.6 (2.9; 4.3)	3.8 (3.2; 4.7)
Serum HDL cholesterol (mmol/L), Median (IQR)	1.3 (1.1; 1.6)	1.2 (1.0; 1.4)
Serum triglyceride (mmol/L), Median (IQR)	1.2 (0.9; 1.6)	1.4 (1.1; 2.2)
Serum fasting glucose (mmol/L), Median (IQR)	5.2 (4.9; 5.5)	5.6 (5.2; 5.9)

^1^Crude n, weighted prevalence, and median. ^2^Winter: November-March; Other season: April–October. Abbreviations: *S-25(OH)D* (serum 25-hydroxyvitamin D), *H2000* (Health 2000), *H2011* (Health 2011), *BMI* (body mass index), *HDL* (high-density lipoprotein), *LDL* (low-density lipoprotein). Missing information: educational status (n = 10), marital status (n = 10), BMI (n = 1), physical activity (n = 31), smoking status (n = 11), alcohol consumption (n = 69), systolic blood pressure (n = 5), diastolic blood pressure (n = 6), total cholesterol (n = 2), serum LDL cholesterol (n = 2), serum HDL cholesterol (n = 2), serum triglyceride (n = 2), serum fasting glucose (n = 2)

**Table 2:** Hazard ratios (HRs) with 95% CIs for type 2 diabetes incidence by S-25(OH)D in H2011 and longitudinal changes in S-25(OH)D (tertiles), n = 3014—non-diabetic participants in both H2000/H2011^1^

	n	Incident cases, % (n)	HR (95% CI)		
			Model 1	Model 2	Model 3
**S-25(OH)D in H2011—all subjects**					
All	3014	7 (214)			
1st (< 62.8)	982	9 (90)	1.37 (1.00–1.88)	1.18 (0.85–1.64)	1.13 (0.81–1.56)
2nd (62.8–71.6)	1002	6 (60)	0.89 (0.61–1.30)	0.82 (0.56–1.21)	0.81 (0.55–1.20)
3rd (> 71.6)	1030	6 (64)	1.00	1.00	1.00
**S-25(OH)D in H2011—subjects with insufficient vitamin D status in H2000**					
All	1708	8 (137)			
1st (< 62.8)	755	11 (77)	1.82 (1.17–2.81)	1.61 (1.04–2.49)	1.61 (1.03–2.51)
2nd (62.8–71.6)	551	7 (37)	1.14 (0.64–2.01)	1.08 (0.61–1.92)	1.10 (0.62–1.95)
3rd (> 71.6)	402	6 (23)	1.00	1.00	1.00
**S-25(OH)D in H2011—subjects with sufficient vitamin D status in H2000**					
All	1306	6 (77)			
1st (< 62.8)	227	6 (13)	0.84 (0.44–1.60)	0.72 (0.36–1.44)	0.60 (0.30–1.23)
2nd (62.8–71.6)	451	5 (23)	0.74 (0.44–1.25)	0.66 (0.39–1.12)	0.63 (0.36–1.11)
3rd (> 71.6)	628	7 (41)	1.00	1.00	1.00
**Changes in S-25(OH)D—all subjects**					
All	3014	7 (214)			
1st (< 13.2)	995	6 (60)	1.30 (0.84–2.01)	1.12 (0.72–1.75)	1.09 (0.70–1.70)
2nd (13.2–26.0)	1024	9 (88)	1.55 (1.10–2.19)	1.42 (1.00**–**2.02)	1.37 (0.96–1.97)
3rd (> 26.0)	995	7 (66)	1.00	1.00	1.00

^1^Crude n, hazard ratios (HRs) with 95% confidence intervals (CIs)

Abbreviations: *S-25(OH)D* (serum 25-hydroxyvitamin D), *H2000* (Health 2000), *H2011* (Health 2011), *BMI* (body mass index).

Model 1: adjusted for age in H2011 (as a continuous variable), sex, baseline S-25(OH)D, and season of blood sampling (winter 2000 and 2011, other season 2000 and 2011, winter 2000 and other season 2011, other season 2000 and winter 2011)

Model 2: adjusted for model 1 + BMI (as a continuous variable)

Model 3: adjusted for model 2 + educational status (low, middle, high), physical activity (physical inactivity, moderate physical activity ≥ 4 h/week, active/vigorous physical activity ≥ 3 h/week), and smoking status (no, yes)

 And some of the current cited references in the published paper are not correct. The correct reference section should read as

**References**

1. Bouillon R, Marcocci C, Carmeliet G, Bikle D, White JH, Dawson-Hughes B, Lips P, Munns CF, Lazaretti-Castro M, Giustina A et al (2019) Skeletal and extraskeletal actions of vitamin D: current evidence and outstanding questions. Endocr Rev 40:1109–1151. 10.1210/er.2018-00126
2. Ghaseminejad-Raeini A, Ghaderi A, Sharafi A, Nematollahi-Sani B, Moossavi M, Derakhshani A, Sarab GA (2023) Immunomodulatory actions of vitamin D in various immune-related disorders: a comprehensive review. Front Immunol 14:950465. 10.3389/fimmu.2023.950465
3. Harrison SR, Li D, Jeffery LE, Raza K, Hewison M (2020) Vitamin D, autoimmune disease and rheumatoid arthritis. Calcif Tissue Int 106:58–75. 10.1007/s00223-019-00577-2
4. WHO (2023) Diabetes. https://www.who.int/health-topics/diabetes/diabetes#tab=tab_1. Accessed 19 December 2024
5. Finnish Diabetes Association (2024) Diabetes in Finland. https://www.diabetes.fi/en/finnish_diabetes_association/diabetes_in_finland. Accessed 10 May 2024
6. Lamberg-Allardt CJE, Outila TA, Kärkkäinen MUM, Rita HJ, Valsta LM (2001) Vitamin D deficiency and bone health in healthy adults in Finland: could this be a concern in other parts of Europe? J Bone Miner Res 16:2066–2073. 10.1359/jbmr.2001.16.11.2066
7. Ministry of Trade and Industry of Finland (2002) FINLEX ® - Säädökset alkuperäisinä: Kauppa- ja teollisuusministeriön asetus… 917/2002. Oikeusministeriö. https://finlex.fi/fi/laki/alkup/2002/20020917. Accessed 19 Dec 2024
8. National Nutrition Council (2010) Valtion ravitsemusneuvottelukunta. D-vitamiinityöryhmän raportti. [Report of vitamin D working group.] In: Finnish. https://www.ruokavirasto.fi/globalassets/teemat/terveytta-edistava-ruokavalio/ravitsemus--ja-ruokasuositukset/erityisohjeet-ja-rajoitukset/d-vitamiiniraportti2010.pdf. Accessed 19 Dec 2024
9. Jääskeläinen T, Itkonen ST, Lundqvist A, Erkkola M, Koskela T, Lakkala K, Dowling KG, Hull GL, Kröger H, Karppinen J et al (2017) The positive impact of general vitamin D food fortification policy on vitamin D status in a representative adult Finnish population: evidence from an 11-y follow-up based on standardized 25-hydroxyvitamin D data. Am J Clin Nutr 105:1512–1520. 10.3945/ajcn.116.151415
10. Paturi M, Tapanainen H, Reinivuo H, Pietinen P editors (2008) The National FINDIET 2007 Survey. In: Finnish, with English abstract, tables, figures and summary. National Public Health Institute. B23/2008. Helsinki: Yliopistopaino. https://www.julkari.fi/bitstream/handle/10024/78088/2008b23.pdf. Accessed 19 Dec 2024
11. Raulio S, Erlund I, Männistö S, Sarlio-Lähteenkorva S, Sundvall J, Tapanainen H, Vartiainen E, Virtanen SM (2017) Successful nutrition policy: improvement of vitamin D intake and status in Finnish adults over the last decade. Eur J Public Health 27:268–273. 10.1093/eurpub/ckw154
12. Valsta L, Kaartinen N, Tapanainen H, Männistö S, Katri Sääksjärvi editors (2018) Nutrition in Finland – The National FinDiet 2017 Survey. In: Finnish, with English abstract. National Institute for Health and Welfare (THL). Report 12/2018. Helsinki, Finland [D4_Julkaistu kehittämis- tai tutkimusraportti taikka -selvitys]. https://www.julkari.fi/handle/10024/137433. Accessed 19 Dec 2024
13. Heistaro S (2008) Methodology report: Health 2000 survey. https://www.julkari.fi/handle/10024/78185. Accessed 19 Dec 2024
14. Lundqvist A, Mäki-Opas T (2016) Health 2011 Survey—Methods [D6]. THL. https://www.julkari.fi/handle/10024/130780. Accessed 19 Dec 2024
15. World Health Organization. (2000) OBESITY: PREVENTING AND MANAGING THE GLOBAL EPIDEMIC. https://iris.who.int/handle/10665/42330
16. Cashman KD, Dowling KG, Škrabáková Z, Gonzalez-Gross M, Valtueña J, De Henauw S, Moreno L, Damsgaard CT, Michaelsen KF, Mølgaard C et al (2016) Vitamin D deficiency in Europe: pandemic? Am J Clin Nutr 10:1033–1044. 10.3945/ajcn.115.120873
17. Centers for Disease Control and Prevention (2020) Certified Total 25-hydroxyvitamin D Procedures. https://www.cdc.gov/clinical-standardization-programs/media/pdfs/2024/04/CDC-Certified-Vitamin-D-Procedures-508.pdf Accessed 19 Dec 2024
18. Binkley N, Sempos CT (2014) Standardizing Vitamin D Assays: The Way Forward. J Bone Miner Res 29:1709–1714
19. Ross AC, Taylor CL, Yaktine AL, Valle HBD (2011) Dietary Reference Intakes for Calcium and Vitamin D. National Academies Press (US). 10.17226/13050
20. Blomhoff R, Andersen R, Arnesen EK, Christensen JJ, Eneroth H, Erkkola M, Gudanaviciene I, Halldórsson ÞI, Höyer-Lund A, Lemming EW (2023) Nordic Nutrition Recommendations 2023: Integrating environmental aspects. Nordic Council of Ministers
21. National Nutrition Council (2014) Kestävää terveyttä ruoasta – kansalliset ravitsemussuositukset. [Sustainable health from food – Finnish Nutrition Recommendations] In: Finnish. Helsinki: PunaMusta Oy. https://www.julkari.fi/handle/10024/150005. Accessed 19 Dec 2024
22. Arffman M, Ilanne-Parikka P, Keskimäki I, Kurkela O, Lindström J, Sund R, Winell K (2020) FinDM database on diabetes in Finland, Discussion paper 19/2020. Finnish Institute for Health and Welfare (THL). https://urn.fi/URN:ISBN:978-952-343-492-9
23. Härkänen T, Karvanen J, Tolonen H, Lehtonen R, Djerf K, Juntunen T, Koskinen S (2016) Systematic handling of missing data in complex study designs – experiences from the Health 2000 and 2011 Surveys. J Appl Stat 43:2772–2790. 10.1080/02664763.2016.1144725
24. Robins JM, Rotnitzky A, Zhao LP (1994) Estimation of regression coefficients when some regressors are not always observed. J Am Stat Assoc 89:846–866. 10.1080/01621459.1994.10476818
25. Tennant PWG, Murray EJ, Arnold KF et al (2021) Use of directed acyclic graphs (DAGs) to identify confounders in applied health research: review and recommendations. Int J Epidemiol 50:620–632. 10.1093/ije/dyaa213
26. Wu F, Juonala M, Pitkänen N, Jula A, Lehtimäki T, Sabin MA, Pahkala K, Hutri-Kähönen N, Kähönen M, Laitinen T et al (2018) Both youth and long-term vitamin D status is associated with risk of type 2 diabetes mellitus in adulthood: a cohort study. Ann Med 50:74–82. 10.1080/07853890.2017.1399446
27. Gagnon C, Lu ZX, Magliano DJ, Dunstan DW, Shaw JE, Zimmet PZ, Sikaris K, Grantham N, Ebeling PR, Daly RM (2011) Serum 25-hydroxyvitamin D, calcium intake, and risk of type 2 diabetes after 5 years. Diabetes Care 34:1133–1138
28. Park SK, Garland CF, Gorham ED, BuDoff L, Barrett-Connor E (2018) Plasma 25-hydroxyvitamin D concentration and risk of type 2 diabetes and pre-diabetes: 12-year cohort study. PLoS ONE 13:e0193070. 10.1371/journal.pone.0193070
29. Zhao N, Zhen D, Zhao Z, Fu S, Guan C, Liu L, Tang X (2022) 25-hydroxyvitamin D and incidence of type 2 diabetes from a Chinese cohort study. J Nutr Sci Vitaminol (Tokyo) 68:8–15. 10.3177/jnsv.68.8
30. Virtanen JK, Hantunen S, Kallio N, Lamberg-Allardt C, Manson JE, Nurmi T, Pihlajamäki J, Uusitupa M, Voutilainen A, Tuomainen T-P (2024) The effect of vitamin D3 supplementation on the incidence of type 2 diabetes in healthy older adults not at high risk for diabetes (FIND): a randomised controlled trial. Diabetologia. 10.1007/s00125-024-06336-9
31. Pittas AG, Dawson-Hughes B, Sheehan P, et al (2019) Vitamin D Supplementation and Prevention of Type 2 Diabetes. N Engl J Med 382:520–530
32. Afzal S, Bojesen SE, Nordestgaard BG (2013) Low 25-hydroxyvitamin D and risk of type 2 diabetes: a prospective cohort study and metaanalysis. Clin Chem 59:381–391. 10.1373/clinchem.2012.193003
33. Contreras-Bolívar V, García-Fontana B, García-Fontana C, Muñoz-Torres M (2021) Mechanisms involved in the relationship between vitamin D and insulin resistance: impact on clinical practice. Nutrients 13:3491. 10.3390/nu13103491
34. Denos M, Mai X-M, Åsvold BO, Sørgjerd EP, Chen Y, Sun Y-Q (2021) Vitamin D status and risk of type 2 diabetes in the Norwegian HUNT cohort study: does family history or genetic predisposition modify the association? BMJ Open Diabetes Res Care 9:e001948. 10.1136/bmjdrc-2020-001948
35. Gao Y, Zheng T, Ran X, Ren Y, Chen T, Zhong L, Yan D, Yan F, Wu Q, Tian H (2018) Vitamin D and incidence of prediabetes or type 2 diabetes: a four-year follow-up community-based study. Dis Markers 2018:1926308. 10.1155/2018/1926308
36. Zheng J-S, Luan J, Sofianopoulou E, Sharp SJ, Day FR, Imamura F, Gundersen TE, Lotta LA, Sluijs I, Stewart ID et al (2020) The association between circulating 25-hydroxyvitamin D metabolites and type 2 diabetes in European populations: a meta-analysis and Mendelian randomisation analysis. PLoS Med 17:e1003394. 10.1371/journal.pmed.1003394
37. Rohold CK, Jørgensen HL, Vojdeman FJ, Madsen CM, Olsen A, Heegaard A-M, Lind BS, Tjønneland A, Schwarz P, Gæde PH (2024) Levels of plasma 25-hydroxy vitamin D and risk of developing type 2 diabetes in a large Danish primary health care population. Acta Diabetol. 10.1007/s00592-024-02368-0
38. Shah VP, Nayfeh T, Alsawaf Y, Saadi S, Farah M, Zhu Y, Firwana M, Seisa M, Wang Z, Scragg R et al (2024) A systematic review supporting the Endocrine Society clinical practice guidelines on vitamin D. J Clin Endocrinol Metab 109:1961–1974. 10.1210/clinem/dgae312
39. Allaoui G, Rylander C, Fuskevåg O-M, Averina M, Wilsgaard T, Brustad M, Jorde R, Berg V (2023) Longitudinal changes in vitamin D concentrations and the association with type 2 diabetes mellitus: the Tromsø Study. Acta Diabetol 60:293–304. 10.1007/s00592-022-02001-y
40. Kaykhaei MA, Khodadoost M, Dashipour AR, Haidari Z, Karimkoshteh A, Sandoughi M (2019) Baseline levels determine magnitude of increment in 25 hydroxy vitamin D following vitamin D3 prescription in healthy subjects. Endocrine 64:378–383. 10.1007/s12020-019-01881-5
41. Zhu K, Hunter M, Hui J, Murray K, James A, Lim EM, Cooke BR, Walsh JP (2022) Longitudinal stability of vitamin D status and its association with bone mineral density in middle-aged Australians. J Endocr Soc 7:bvac187. 10.1210/jendso/bvac187
42. McCourt AF, Mulrooney SL, O’Neill GJ, O’Riordan ED, O’Sullivan AM (2022) Postprandial 25-hydroxyvitamin D response varies according to the lipid composition of a vitamin D3 fortified dairy drink. Int J Food Sci Nutr 73:396–406. 10.1080/09637486.2021.1984400
43. Schafer AL, Napoli N, Lui L, Schwartz AV, Black DM, Study of Osteoporotic Fractures (2014) Serum 25‐hydroxyvitamin D concentration does not independently predict incident diabetes in older women. Diabet Med 31:564–569. 10.1111/dme.12368
44. Drincic AT, Armas LAG, Van Diest EE, Heaney RP (2012) Volumetric dilution, rather than sequestration best explains the low vitamin D status of obesity. Obesity 20:1444–1448. 10.1038/oby.2011.404
45. Tobias DK, Luttmann-Gibson H, Mora S, Danik J, Bubes V, Copeland T, LeBoff MS, Cook NR, Lee I-M, Buring JE et al (2023) Association of body weight with response to vitamin D supplementation and metabolism. JAMA Netw Open 6:e2250681. 10.1001/jamanetworkopen.2022.50681
46. Jääskeläinen T, Knekt P, Marniemi J, Sares-Jäske L, Männistö S, Heliövaara M, Järvinen R (2013) Vitamin D status is associated with sociodemographic factors, lifestyle and metabolic health. Eur J Nutr 52:513–525. 10.1007/s00394-012-0354-0
47. Sempos CT, Vesper HW, Phinney KW, Thienpont LM, Coates PM, Vitamin D Standardization Program (VDSP) (2012) Vitamin D status as an international issue: national surveys and the problem of standardization. Scand J Clin Lab Invest Suppl 243:32–40. 10.3109/00365513.2012.681935
48. Bullard KM, Cowie CC, Lessem SE, Saydah SH, Menke A, Geiss LS, Orchard TJ, Rolka DB, Imperatore G (2018) Prevalence of diagnosed diabetes in adults by diabetes type—United States, 2016. MMWR Morb Mortal Wkly Rep 67:359–361. 10.15585/mmwr.mm6712a2
49. Wang X, Kattan MW (2020) Cohort studies. Chest 158:S72–S78. 10.1016/j.chest.2020.03.014

The original article has been corrected.

