# Supplementary material for: Vitamin D status and type 2 diabetes incidence in Finnish adults—a longitudinal survey and register-based study using standardized serum 25-hydroxyvitamin D data
Source: Eur J Nutr. 2026 Jan 24;65(1):34. doi: 10.1007/s00394-025-03889-2 (PMC12831685; doi:10.1007/s00394-025-03889-2)
Supplement: Supplementary file 1 — Supplementary Material 1 [file 394_2025_3889_MOESM1_ESM.doc]

**Supplementary Information**


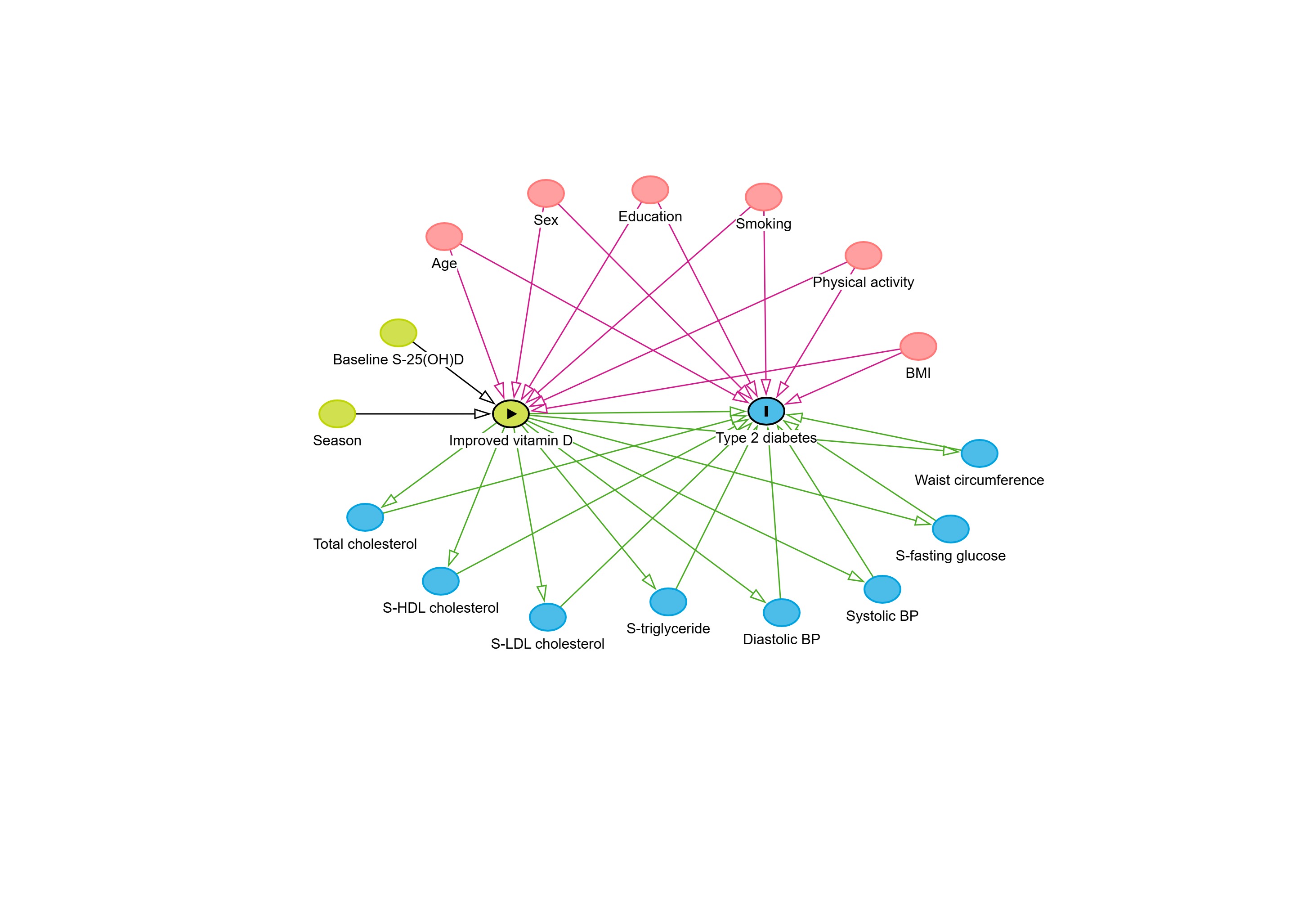


**Fig. S1** Directed acyclic graph (DAG) on improved vitamin D (S-25(OH)D in H2011, exposure variable) and type 2 diabetes (outcome variable). Legend: The variable in green and with the “►” symbol inside the circle is the exposure variable; the variable in blue and with the letter “I” inside the circle is the outcome variable; variables in green are the antecedents of the exposure variable; those in blue are the antecedents of the outcome variable; and those in red are the antecedents of the outcome and exposure variables i.e., confounders. Black arrows are non-causal and unbiased paths; green arrows are causal paths between the exposure variable and outcome variable or antecedents; red arrows are biased paths.

**Table S1** VDSP-standardized S-25(OH)D concentrations in H2011 participants with one time point (in H2000 or H2011) and two time points (in both H2000 and H2011) measurements1

|  | One time point measurement | | Two time point measurements |
| --- | --- | --- | --- |
|  | H2000 | H2011 | H2000 and H2011 |
| No prevalent diabetes | n=4652 | n=3759 | n=3014 |
| S-25(OH)D) in 2011, Median (IQR) | - | 67.2 (60.0; 74.8) | 67.2 (60.0; 74.8) |
| S-25(OH)D) in 2000, Median (IQR) | 46.6 (37.1; 57.4) | - | 47.6 (37.1; 57.4) |
| Including prevalent diabetes | n=5228 | n=4102 | n=3328 |
| S-25(OH)D) in 2011, Median (IQR) | - | 67.2 (60.0; 74.8) | 67.2 (60.0; 74.3) |
| S-25(OH)D) in 2000, Median (IQR) | 46.6 (36.1; 56.7) | - | 47.6 (37.1; 57.4) |

^1^Crude n, and weighted median. Abbreviations: *VDSP* (Vitamin D Standardization Program), *S-25(OH)D* (serum 25-hydroxyvitamin D), *H2000* (Health 2000), *H2011* (Health 2011)

**Table S2** VDSP-standardized S-25(OH)D concentrations at H2000 and H2011 according to participants’ characteristics (at H2000), n=3014 – non-diabetic participants in both H2000/H20111

| Sociodemographic variables |  | S-25(OH)D (nmol/L), Median (IQR) | |
| --- | --- | --- | --- |
|  | Prevalences, % (n) | H2000 | H2011 |
| All | 100 (3014) | 47.6 (37.1; 57.4) | 67.2 (60.0; 74.8) |
| Age (years)  30-39  40-49  50-59  60-69  >70 | 29 (855)  28 (861)  25 (772)  14 (406)  5 (120) | 43.4 (34.0; 54.4)  45.5 (36.1; 56.7)  48.7 (39.2; 58.9)  51.8 (42.4; 60.4)  55.2 (42.4; 61.9) | 67.2 (59.5; 74.3)  67.2 (59.5; 74.8)  67.7 (61.1; 74.8)  67.7 (61.1; 74.3)  66.6 (58.4; 73.2) |
| Sex  Men  Women | 45 (1325)  55 (1689) | 47.6 (38.2; 58.2)  46.6 (37.1; 56.7) | 67.2 (60.6; 75.4)  67.2 (60.0; 74.3) |
| Season of blood sampling^2^  Winter 2000 and 2011  Other seasons 2000 and 2011  Winter 2000 and other season 2011  Other season 2000 and winter 2011 | 28 (862)  27 (801)  37 (1104)  8 (247) | 44.5 (35.0; 54.4)  52.9 (42.4; 61.9)  44.5 (35.0; 55.9)  53.7 (42.4; 61.1) | 65.0 (58.4; 72.1)  68.8 (61.7; 77.0)  68.3 (61.1; 75.4)  65.5 (57.8; 72.1) |
| Educational status  Low  Middle  High | 30 (827)  35 (1055)  35 (1122) | 48.7 (38.2; 58.9)  46.6 (36.1; 55.9)  47.6 (37.1; 57.4) | 67.2 (60.0; 74.3)  67.2 (60.0; 74.8)  67.2 (60.0; 74.8) |
| Marital status  Married or cohabitating  Others | 77 (2330)  23 (674) | 47.6 (38.2; 57.4)  46.6 (36.1; 56.7) | 67.2 (60.0; 74.8)  66.6 (59.5; 74.8) |
| Lifestyle variables | | | |
| BMI (kg/m^2^)  <18.5 (underweight)  18.5–24.9 (normal weight)  25.0–29.9 (overweight  ≥30.0 (obesity) | 1 (16)  42 (1283)  40 (1204)  17 (510) | 41.3 (35.0; 70.8)  48.7 (38.2; 58.9)  46.6 (37.1; 57.4)  44.5 (36.1; 55.2) | 68.8 (51.2; 77.6)  68.3 (61.1; 76.5)  66.6 (60.0; 74.3)  65.0 (57.8; 71.6) |
| Physical activity  Inactive physical activity  Moderate physical activity ≥4 h/week  Active/vigorous physical activity ≥3 h/week | 22 (631)  56 (1694)  22 (658) | 42.4 (34.0; 53.7)  48.7 (38.2; 58.2)  49.7 (39.2; 58.9) | 65.5 (58.4; 73.2)  67.2 (60.6; 74.8)  68.8 (62.2; 76.5) |
| Current smoking  No  Yes | 74 (2254)  26 (749) | 48.7 (38.2; 58.9)  43.4 (35.0; 54.4) | 67.7 (60.6; 74.8)  65.5 (58.4; 73.7) |
| Alcohol consumption  No  Yes | 14 (399)  86 (2546) | 46.6 (36.1; 55.2)  47.6 (37.1; 58.2) | 65.5 (58.4; 72.1)  67.7 (60.6; 74.8) |
|  |  |  |  |

^1^Crude n, weighted prevalence, and median.^2^Winter: November-March; Other season: April-October. Abbreviations: *VDSP* (Vitamin D Standardization Program), *S-25(OH)D* (serum 25-hydroxyvitamin D), *H2000* (Health 2000), *H2011* (Health 2011), *BMI* (body mass index). Missing information: educational status (n=10), marital status (n=10), BMI (n=1), physical activity (n=31), smoking status (n=11), alcohol consumption (n=69)

**Table S3** Hazard ratios (HRs) with 95% CIs for type 2 diabetes incidence by S-25(OH)D in H2011 (tertiles) – sensitivity analysis: stratified by BMI, n=3014 – non-diabetic participants in both H2000/H2011^1^

| S-25(OH)D in H2011, nmol/L | n | Incident cases, % (n) | HR (95% CI) | | | |
| --- | --- | --- | --- | --- | --- | --- |
|  |  |  | Model 1 | Model 2 | Model 3 |  |
| All subjects | | | | | | |
| All  1^st^ (<62.8)  2^nd^ (62.8-71.6)  3^rd^ (>71.6) | 3014  982  1002  1030 | 7 (214)  9 (90)  6 (60)  6 (64) | 1.37 (1.00-1.88)  0.89 (0.61-1.30)  1.00 | 1.18 (0.85-1.64)  0.82 (0.56-1.21)  1.00 | 1.13 (0.81-1.56)  0.81 (0.55-1.20)  1.00 |  |
| Subjects with BMI <25 kg/m^2^ | | | | | | |
| All  1^st^ (<62.8)  2^nd^ (62.8-71.6)  3^rd^ (>71.6) | 1300  377  424  499 | 3 (38)  3 (11)  4 (14)  3 (13) | 1.25 (0.55-2.85)  1.27 (0.59-2.72)  1.00 | 1.25 (0.55-2.85)  1.27 (0.59-2.72)  1.00 | 1.07 (0.46-2.48)  1.22 (0.57-2.62)  1.00 |  |
| Subjects with BMI 25-29.9 kg/m^2^ | | | | | | |
| All  1^st^ (<62.8)  2^nd^ (62.8-71.6)  3^rd^ (>71.6) | 1205  397  404  404 | 8 (96)  11 (41)  7 (28)  7 (27) | 1.59 (0.96-2.65)  1.02 (0.59-1.75)  1.00 | 1.59 (0.96-2.65)  1.02 (0.59-1.75)  1.00 | 1.59 (0.94-2.68)  1.03 (0.59-1.77)  1.00 |  |
| Subjects with BMI ≥30 kg/m^2^ | | | | | | |
| All  1^st^ (<62.8)  2^nd^ (62.8-71.6)  3^rd^ (>71.6) | 509  208  174  127 | 8 (80)  7 (38)  4 (18)  5 (24) | 0.87 (0.52-1.46)  0.49 (0.26-0.93)  1.00 | 0.87 (0.52-1.46  0.49 (0.26-0.93)  1.00 | 0.78 (0.45-1.33)  0.46 (0.24-0.88)  1.00 |  |

^1^Crude n, hazard ratios (HRs) with 95% confidence intervals (CIs).
Abbreviations: *S-25(OH)D* (serum 25-hydroxyvitamin D), *H2000* (Health 2000), *H2011* (Health 2011), *BMI* (body mass index).
Model 1: adjusted for age in H2011 (as a continuous variable), sex, baseline S-25(OH)D, and season of blood sampling (winter 2000 and 2011, other season 2000 and 2011, winter 2000 and other season 2011, other season 2000 and winter 2011)

Model 2: adjusted for model 1 + BMI (as a continuous variable)

Model 3: adjusted for model 2 + educational status (low, middle, high), physical activity (physical inactivity, moderate physical activity ≥4 h/week, active/vigorous physical activity ≥3 h/week), and smoking status (no, yes)

**Table S4** Tertiles of longitudinal changes in S-25(OH)D in relation to S-25(OH)D concentrations and vitamin D status at H2000 (2 cut-offs, categorized variable) (crude n, weighted median), N=3014 – non-diabetic participants in both H2000/H2011 who were followed up for over 8 years (i.e., 2011-2019) 1

| Changes in  S-25(OH)D tertiles (nmol/L) |  | | | Baseline vitamin D status (<50) | | Baseline vitamin D status (≥50) | |
| --- | --- | --- | --- | --- | --- | --- | --- |
|  | Prevalences, % (n) | S-25(OH)D  at H2000,  Median (IQR) | Changes in  S-25(OH)D, Median (IQR) | Prevalences, % (n) | Changes in  S-25(OH)D, Median (IQR) | Prevalence, % (n) | Changes in  S-25(OH)D, Median (IQR) |
| All  1^st^ (<13.2)  2^nd^ (13.2-26.0)  3^rd^ (>26.0) | 3014  33 (995)  34 (1024)  33 (995) | 47.6 (37.1; 57.4)  58.2 (50.8; 65.6)  47.6 (40.3; 55.2)  36.1 (29.7; 43.4) | 19.2 (9.7; 29.6)  5.2 (-0.3; 9.6)  19.1 (16.3; 22.5)  34.3 (29.7; 40.3) | 1708  13 (227)  36 (615)  51 (866) | 26.3 (18.0; 34.8)  7.5 (3.2; 10.8)  20.1 (16.7; 22.9)  34.6 (30.1; 40.7) | 1306  58 (768)  32 (409)  10 (129) | 10.8 (2.9; 18.4)  4.1 (-1.3; 8.8)  18.2 (15.5; 21.1) 31.4 (27.9; 37.3) |

^1^Crude n, weighted prevalence, and median. Abbreviations: *S-25(OH)D* (serum 25-hydroxyvitamin D), *H2000* (Health 2000), *H2011* (Health 2011)

**Table S5** Hazard ratios (HRs) with 95% CIs for type 2 diabetes incidence by longitudinal changes in S-25(OH)D (tertiles), n=3014 – non-diabetic participants in both H2000/H2011^1^

| Changes in S-25(OH)D, nmol/L | n | Incident cases, % (n) | HR (95% CI) | | | |
| --- | --- | --- | --- | --- | --- | --- |
|  |  |  | Model 1 | Model 2 | Model 3 |  |
| All subjects | | | | | |  |
| All  1^st^ (<13.2)  2^nd^ (13.2-26.0)  3^rd^ (>26.0) | 3014  995  1024  995 | 7 (214)  6 (60)  9 (88)  7 (66) | 1.30 (0.84-2.01)  1.55 (1.10-2.19)  1.00 | 1.12 (0.72-1.75)  1.42 (1.00**-**2.02)  1.00 | 1.09 (0.70-1.70)  1.37 (0.96-1.97)  1.00 |  |
| Subjects with insufficient vitamin D status in H2000 | | | | | |  |
| All  1^st^ (<13.2)  2^nd^ (13.2-26.0)  3^rd^ (>26.0) | 1708  227  615  866 | 8 (137)  8 (18)  10 (61)  7 (58) | 1.27 (0.71-2.27)  1.62 (1.12-2.36)  1.00 | 1.15 (0.65-2.05)  1.54 (1.07-2.23)  1.00 | 1.17 (0.65-2.09)  1.54 (1.06-2.24)  1.00 |  |
| Subjects with sufficient vitamin D status in H2000 | | | | | |  |
| All  1^st^ (<13.2)  2^nd^ (13.2-26.0)  3^rd^ (>26.0) | 1306  768  409  129 | 6 (77)  6 (42)  7 (27)  6 (8) | 0.97 (0.43-2.20)  0.96 (0.44-2.11)  1.00 | 0.68 (0.29-1.60)  0.73 (0.33-1.64)  1.00 | 0.66 (0.28-1.56)  0.72 (0.32-1.63)  1.00 |  |

^1^Crude n, hazard ratios (HRs) with 95% confidence intervals (CIs).
Abbreviations: *S-25(OH)D* (serum 25-hydroxyvitamin D), *H2000* (Health 2000), *H2011* (Health 2011), *BMI* (body mass index).
Model 1: adjusted for age in H2011 (as a continuous variable), sex, baseline S-25(OH)D, and season of blood sampling (winter 2000 and 2011, other season 2000 and 2011, winter 2000 and other season 2011, other season 2000 and winter 2011)

Model 2: adjusted for model 1 + BMI (as a continuous variable)

Model 3: adjusted for model 2 + educational status (low, middle, high), physical activity (physical inactivity, moderate physical activity ≥4 h/week, active/vigorous physical activity ≥3 h/week), and smoking status (no, yes)
